# Supplementary material for: Spread spectrum SERS allows label-free detection of attomolar neurotransmitters
Source: Nat Commun. 2021 Jan 8;12:159. doi: 10.1038/s41467-020-20413-8 (PMC7794485; doi:10.1038/s41467-020-20413-8)
Supplement: Supplementary file 1 — Supplementary Information [file 41467_2020_20413_MOESM1_ESM.pdf]

Supplementary Information

Spread Spectrum SERS allows label-free detection of attomolar neurotransmitters

Wonkyoung Lee[1, 2,3], *Byoung-Hoon Kang*[1,2], *Hyunwoo Yang*[1,2], *Moonseong Park*[1], *Ji Hyun Kwak*[1], *Taerin Chung*[1], *Yong Jeong*[1,2], *Bong Kyu Kim*[3], *and Ki-Hun Jeong*[1,2]*

[1]Department of Bio and Brain Engineering, Korea Advanced Institute of Science and Technology (KAIST), 291 Daehak-ro, Yuseong-gu, Daejeon 305-701, Republic of Korea

[2]KAIST Institute for Health Science and Technology (KIHST), KAIST, 291 Daehak-ro, Yuseong-gu, Daejeon 305-701, Republic of Korea

[3]Network Research Division, Electronics and Telecommunications Research Institute (ETRI), 218 Gajeong-ro, Yuseong-gu, Daejeon, 350-700, Republic of Korea

*Corresponding author: kjeong@kaist.ac.kr

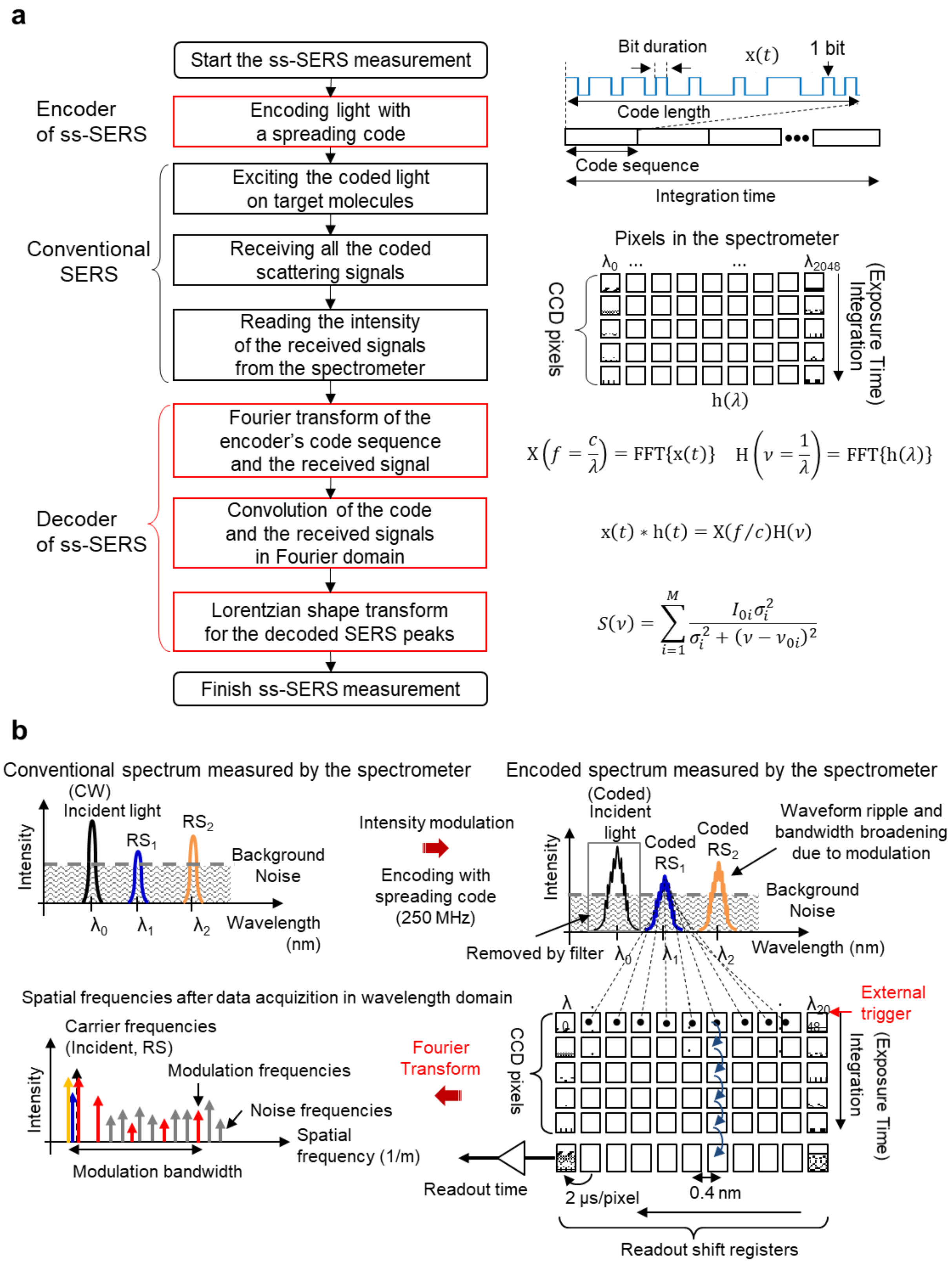

a
Start the ss-SERS measurement
Encoder of ss-SERS
Encoding light with a spreading code
Conventional SERS
Exciting the coded light on target molecules
Receiving all the coded scattering signals
Reading the intensity of the received signals from the spectrometer
Decoder of ss-SERS
Fourier transform of the encoder's code sequence and the received signal
Convolution of the code and the received signals in Fourier domain
Lorentzian shape transform for the decoded SERS peaks
Finish ss-SERS measurement
Bit duration
$x(t)$
1 bit
Code length
Code sequence
Integration time
Pixels in the spectrometer
λ_0
λ_{2048}
CCD pixels
Integration (Exposure Time)
$h(\lambda)$
$X\left(f=\frac{c}{\lambda}\right)=FFT\{x(t)\}$
$H\left(\nu=\frac{1}{\lambda}\right)=FFT\{h(\lambda)\}$
$x(t)*h(t)=X(f/c)H(\nu)$
$S(\nu)=\sum_{i=1}^{M}\frac{I_{0i}\sigma_i^2}{\sigma_i^2+(\nu-\nu_{0i})^2}$
b
Conventional spectrum measured by the spectrometer
(CW) Incident light
RS_1
RS_2
Background Noise
Intensity
λ_0 λ_1 λ_2
Wavelength (nm)
Intensity modulation
Encoding with spreading code (250 MHz)
Encoded spectrum measured by the spectrometer
(Coded) Incident light
Coded RS_1
Coded RS_2
Waveform ripple and bandwidth broadening due to modulation
Background Noise
Removed by filter
Wavelength (nm)
Spatial frequencies after data acquizition in wavelength domain
Carrier frequencies (Incident, RS)
Modulation frequencies
Noise frequencies
Spatial frequency (1/m)
Modulation bandwidth
Fourier Transform
CCD pixels
External trigger
λ_0
λ_{2048}
Integration (Exposure Time)
Readout time
2 μs/pixel
0.4 nm
Readout shift registers

Supplementary Figure 1. ss-SERS decoding process. a ss-SERS measurement procedure. ① Encode CW light from a laser beam with a spreading code of PN code using an intensity modulator in the excitation light encoder. The intensity modulator controlled by output voltages of a function generator converts the polarized CW light into coded light. ② Launch the coded sequences into target molecules on SERS substrates using a fiber bundle-based Raman probe. ③ Receive mixed signals with coded SERS signals, coded Rayleigh scattering signals, coded fluorescence signals, and various system noises using the fiber bundle-based Raman probe and a spectrometer for an integration time, where the coded Rayleigh scattering signals are removed by the notch filter within the Raman probe. ④ Read the pixel values corresponding to the intensities of the received signals from the spectrometer. The procedures from ② to ④ are common to the conventional SERS. The decoding process for restoring final SERS signals through the correlation process between the detected signals and the same PN code are composed of ⑤, ⑥ and ⑦. ⑤ Get Fourier transform of the code sequence and the received signals for the integration time. ⑥ Multiply the fast Fourier transform (FFT) of detected signals and FFT of the PN code for convolution calculation of two functions (Laplace transform). ⑦ Transforms the convolution result into the conventional Raman spectrum of Lorentzian line shape using intensities, center positions, and standard deviations of ss-SERS peaks using the equation given by

$$S(\nu) = \sum_{i=1}^{M} \frac{I_{0i}\sigma_i^2}{\sigma_i^2 + (\nu - \nu_{0i})^2}$$

where, I_{0i} and ν_{0i} are intensity and Raman shift position of ith SERS peak. σ_i means standard deviation associated with Raman line width. **b** Data acquisition from the spectrometer for the Fourier transform-based convolution in the decoding process of the ss-SERS. Unlike conventional

SERS, the frequency components of the amplitude-modulated SERS signals are dispersed by a grating device and detected by each pixel of CCD of the spectrometer as wavelength components and accumulated for a preset integration time. In particular, CMOS and CCD line cameras can be utilized for this method, however, CCDs operating in the global shutter mode facilitate in implementation of the ss-SERS. To synchronize the start time of the CCD exposure with the first bit of the spreading code, the spectrometer triggers using the external trigger signal of the function generator, which sends a voltage signal of the code pattern to the modulator. The stop time of the CCD exposure is determined by the preset integration time. The output signals of the spectrometer are converted into spatial frequencies by the Fourier transform.

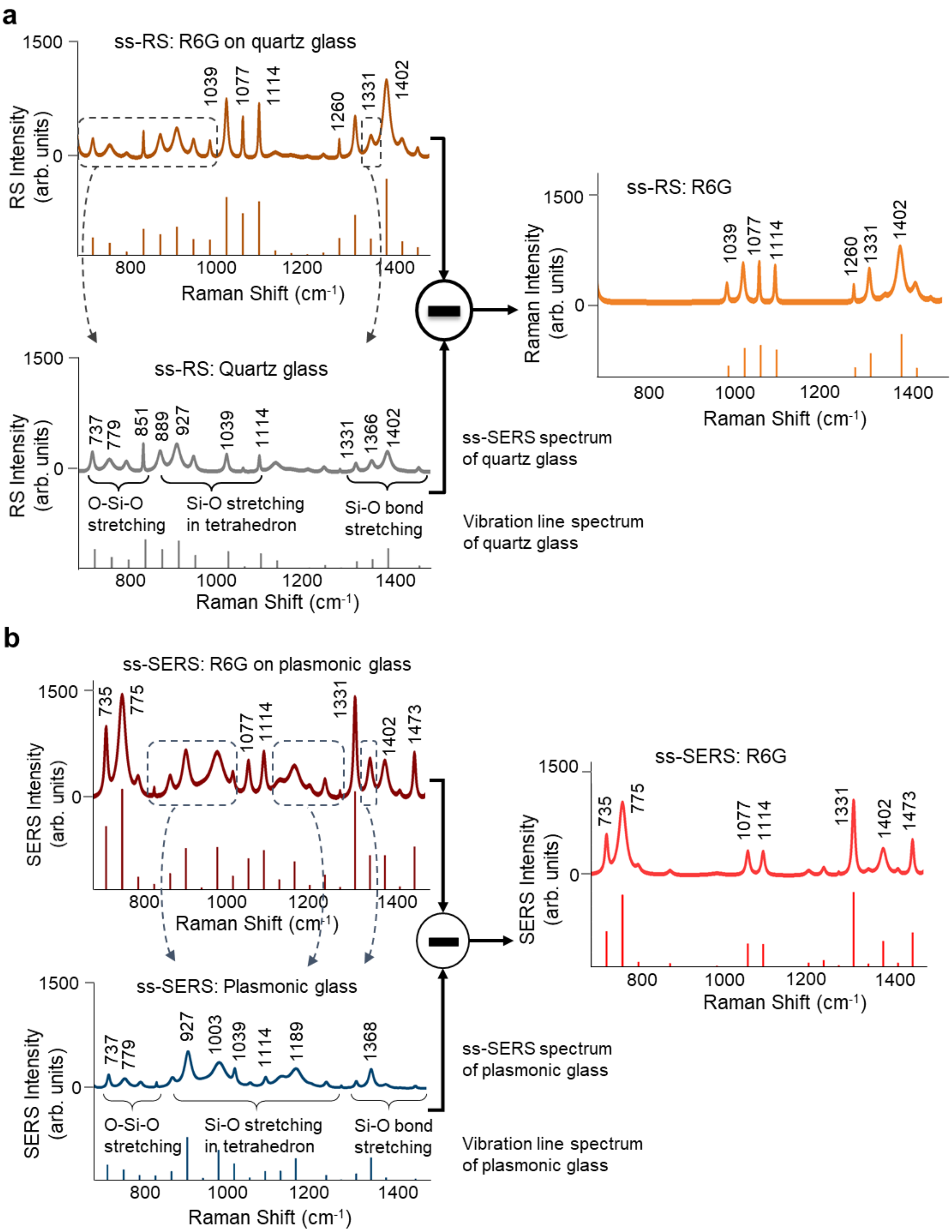
a
ss-RS: R6G on quartz glass
RS Intensity (arb. units)
1500
0
1039
1077
1114
1260
1331
1402
800
1000
1200
1400
Raman Shift (cm-1)
ss-RS: Quartz glass
737
779
851
889
927
1039
1114
1331
1366
1402
O-Si-O stretching
Si-O stretching in tetrahedron
Si-O bond stretching
ss-SERS spectrum of quartz glass
Vibration line spectrum of quartz glass
ss-RS: R6G
Raman Intensity (arb. units)
b
ss-SERS: R6G on plasmonic glass
SERS Intensity (arb. units)
735
775
1077
1114
1331
1402
1473
ss-SERS: Plasmonic glass
737
779
927
1003
1039
1114
1189
1368
O-Si-O stretching
Si-O stretching in tetrahedron
Si-O bond stretching
ss-SERS spectrum of plasmonic glass
Vibration line spectrum of plasmonic glass
ss-SERS: R6G

Supplementary Figure 2. The calibrated ss-RS and ss-SERS spectra. a The calibrated ss-RS spectra are obtained by subtracting the ss-RS signals of quartz glass from the ss-RS signals of R6G on quartz glass. **b** The calibrated ss-SERS spectra are obtained by subtracting the ss-SERS signals of plasmonic glass from the ss-SERS signals of R6G on plasmonic glass.

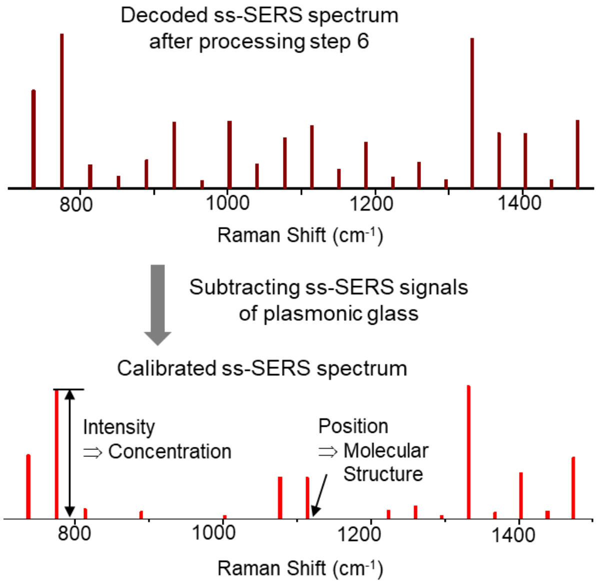

Peak position (ν_{0i})	Intensity (I_{0i})	Standard deviation (σ_i)
736	11852	4.0
775	23727	10.3
813	1888	6.2
889	1396	7.0
1077	7804	5.0
1114	7659	4.7
1223	1553	7.5
1260	2412	5.1
1296	721	1.3
1331	24567	4.0
1367	1250	1.0
1403	8599	9.2
1438	1494	4.7
1473	11403	3.7
1508	13835	2.4
1543	1532	10.4

Lorentzian line shape function

$$S(\nu) = \sum_{i=1}^{M} \frac{I_{0i}\sigma_i^2}{\sigma_i^2 + (\nu - \nu_{0i})^2}$$

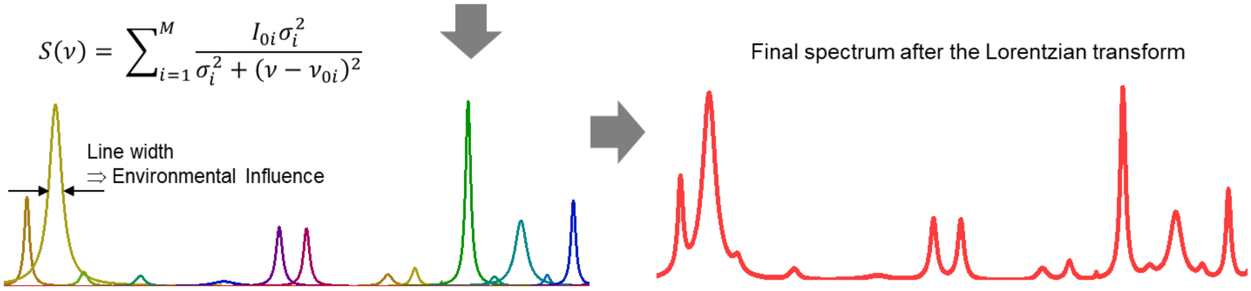

Supplementary Figure 3. Detail description of the Lorentzian line shape transformation in the case of ss-SERS spectrum for R6G 5 mM. Lorentzian Transformation is a fitting procedure, where spectral peaks such as the Raman peaks are fitted to a combination of the Lorentzian line shapes with different widths. After obtaining the peak position (ν_{0i}) and intensity (I_{0i}) values from the decoded spectra (ss-SERS), and repeatedly measuring 5 or more times to calculate the standard deviation (σ_i) of the peak intensity, ss-SERS spectra of the Lorentzian line shape are finally obtained by substituting these parameters into the Lorentzian line shape function. The peak width and the final line shape are obtained by performing Lorentzian curve fitting with parameters such as peak positions, peak intensities, and standard deviation of peak intensities measured from the decoded SERS spectra.

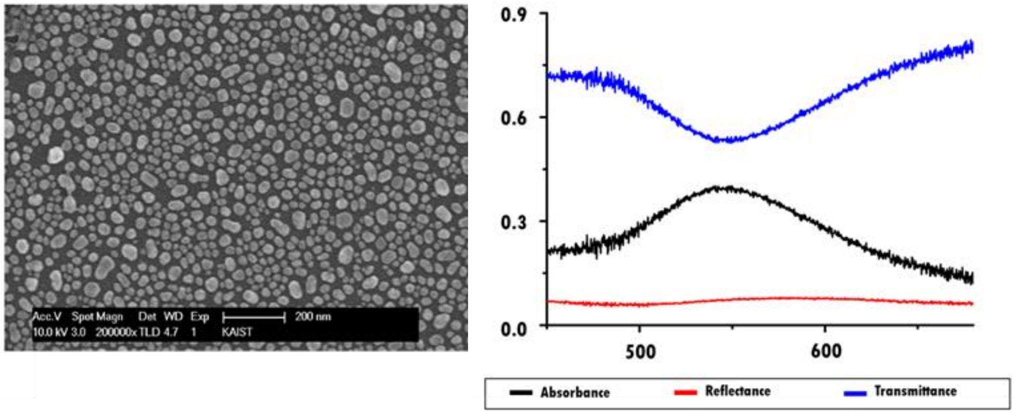

Supplementary Figure 4. Nanogap-rich Au nanoislands on quartz glass as a SERS substrate. The scanning electron microscope (SEM) image on the right side clearly shows nano-gap-rich palsmonic nanoislands on quartz glass, which secure generation of intensity localized electromagnetic field within plasmonic hot spots for SERS.

a

Degree (n)	Primitive Polynomial	Degree (n)	Primitive Polynomial
		11	$x^{11} + x^2 + 1$
2	$x^2 + x + 1$	12	$x^{12} + x^6 + x^4 + x + 1$
3	$x^3 + x + 1$	13	$x^{13} + x^4 + x^3 + 1$
4	$x^4 + x + 1$	14	$x^{14} + x^{10} + x^6 + x + 1$
5	$x^5 + x^2 + 1$	15	$x^{15} + x + 1$
6	$x^6 + x + 1$	16	$x^{16} + x^{12} + x^3 + x + 1$
7	$x^7 + x^3 + 1$	17	$x^{17} + x^3 + 1$
8	$x^8 + x^4 + x^3 + x^2 + 1$	18	$x^{18} + x^7 + 1$
9	$x^9 + x^4 + 1$	19	$x^{19} + x^5 + x^2 + x + 1$
10	$x^{10} + x^3 + 1$	20	$x^{20} + x^3 + 1$

b

Degree (n)	Non-Primitive Polynomial	Degree (n)	Non-Primitive Polynomial
2	$x^2 + 1$	8	$x^8 + x^7 + x^4 + x^2 + 1$
3	$x^3 + x^2 + 1$	9	$x^9 + x^7 + x^3 + x^2 + 1$
4	$x^4 + x^3 + x^2 + 1$	10	$x^{10} + x^5 + x^2 + 1$
5	$x^5 + x + 1$	11	$x^{11} + x^3 + x + 1$
6	$x^6 + x^5 + x^4 + x^3 + x + 1$	12	$x^{12} + x^7 + x^2 + 1$
7	$x^7 + x^6 + x^3 + x^2 + 1$	13	$x^{13} + x^4 + x^2 + 1$

c

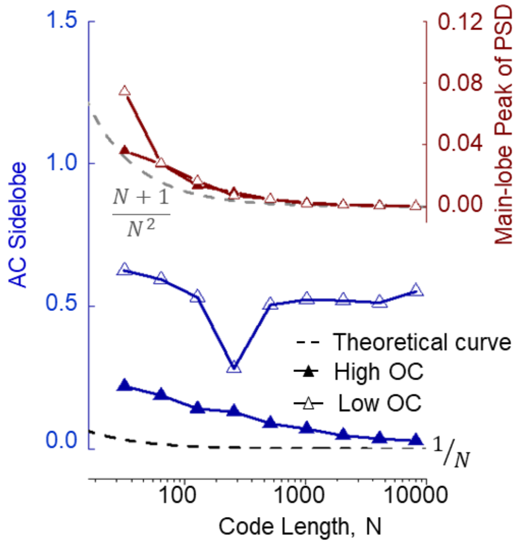

Supplementary Figure 5. Primitive polynomials and autocorrelation sidelobes for generation of PN sequences compared to non-primitive polynomials. a Primitive polynomials of degrees from 2 to 20. PN code sequences are generated by using a custom designed MATLAB script based

on PN sequence generator module with the primitive polynomials in MATLAB libraries. A PN code sequence of high orthogonality was generated by using linear feedback shifter register (LFSR) with maximum cycle length based on an irreducible primitive polynomial. **b** Non-primitive polynomials of degrees from 2 to 13. A PN code sequence of low orthogonality to reduce implementation complexity in other applications except for cryptography and error detection and correction may be generated by LFSR with non-maximum cycle length based on non-primitive polynomial. **c** The autocorrelation (AC) sidelobe and the main-lobe peak for high and low orthogonal codes depending on the code length. The calculated results from the anti-noise performance factors clearly show that in case of high orthogonal code, the autocorrelation sidelobe and the main-lobe peak approach the theoretical curve as the code length increases, whereas the autocorrelation sidelobe of the low orthogonal PN code hardly decreases after the code length exceeds 254 bits, and the main-lobe peak increases as opposed to the theoretical curve after over 128 code length.

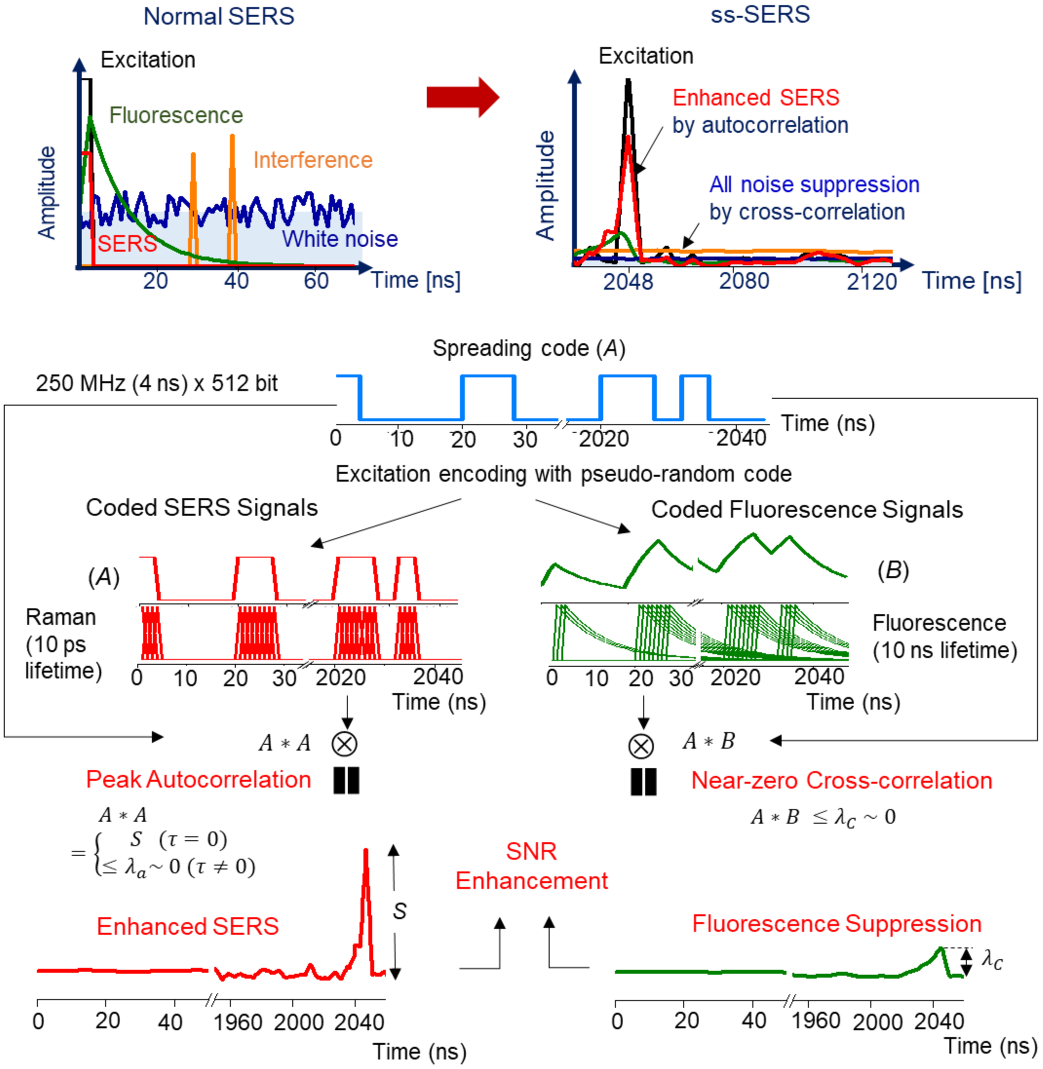

Supplementary Figure 6. The suppression mechanism of fluorescence background noise in the ss-SERS at 250 MHz modulation frequency. By maximizing the randomness of the orthogonal code, the unwanted signal (noise) that is not the same pattern as the orthogonal code spreads out near zero in the decoding process. The novelty of this work is to improve signal-to-noise ratio (SNR) of SERS/Raman signals in noisy environment by applying peak autocorrelation and near-zero cross-correlation to SERS/Raman detection.

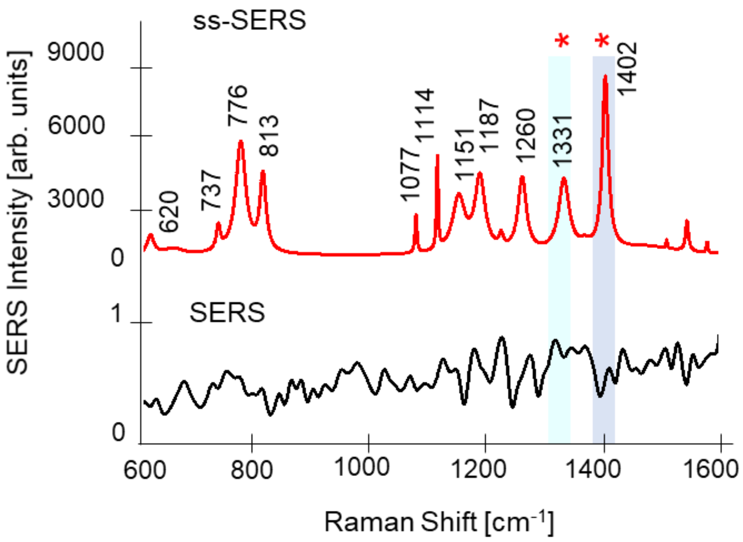

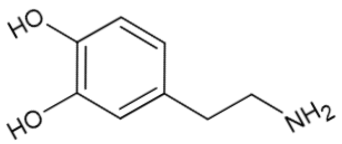

Peaks (cm^{-1})	Vibrational Assignment
737 ~ 776	δ(CH)
813	τ(NH)
1077	v(C-C-N), ω(CH)
1114	τ(CH), τ(NH), τ(CN)
1151 ~ 1187	ρ(OH), ρ(CH), ω(CH)
1260 ~ 1331	ρ(CH), τ(CH)
1402	ω(CH), τ(NH)

Supplementary Figure 7. The measured ss-SERS spectrum and vibrational assignments of dopamine in the ss-SERS, compared with the SERS. The ss-SERS peak intensity of dopamine at 1402 cm^{-1} corresponding to CH wagging and NH twisting is observed to be maximized more than one thousand times than the SERS at 1 mM in saline solution. The main vibrational modes for dominant peaks around 737, 776, 813, 1077, 1114, 1151, 1187, 1260, 1331, 1402 cm^{-1} are also summarized. (The output power of the laser: 25 mW, the power at the sample: 1 mW, accumulation time: 10 sec)

δ, bending; τ, twisting; ω, wagging; v, stretching; ρ, rocking.

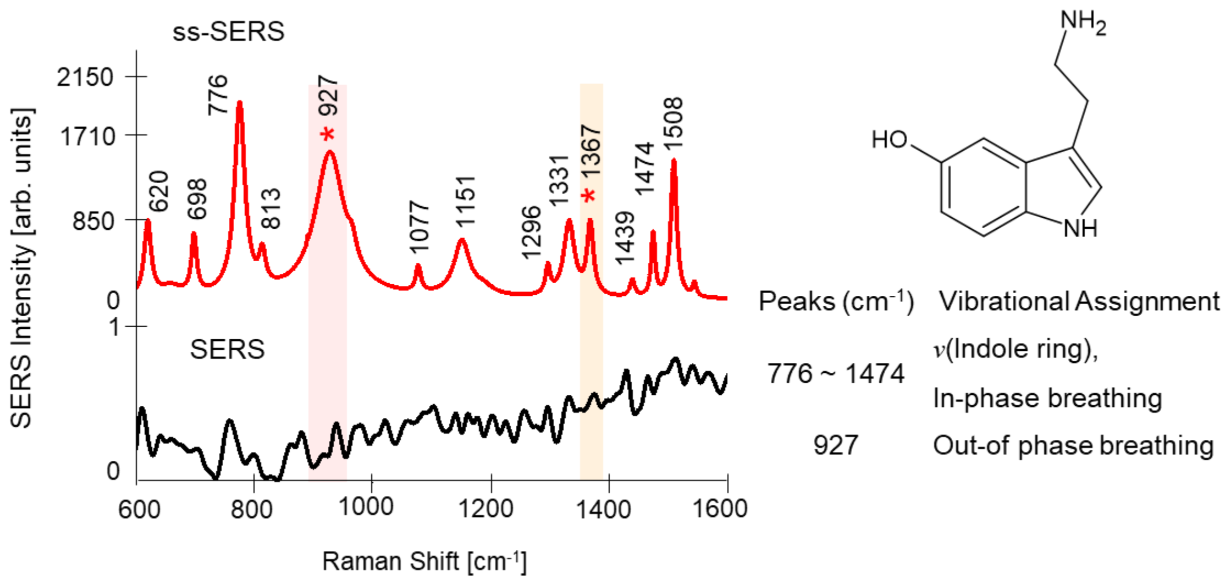

Supplementary Figure 8. The measured ss-SERS spectrum and vibrational assignments of serotonin in the ss-SERS, compared with the SERS. The ss-SERS peak intensity of serotonin at 927 cm^{-1} corresponding to out of phase breathing of the indole ring is measured to be maximized more than one thousand times than the SERS at 1 mM in saline solution. (The output power of the laser: 25 mW, the power at the sample: 1 mW, accumulation time: 10 sec)

v, stretching.

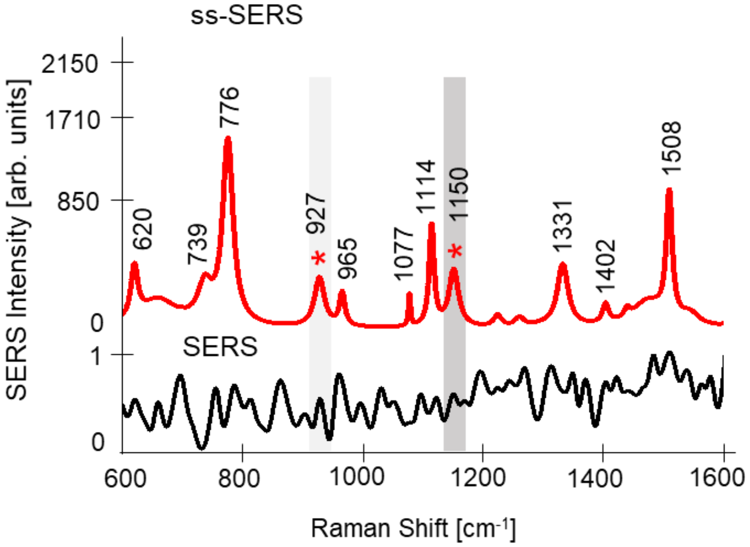

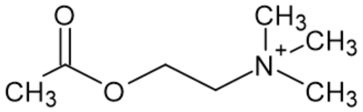

Peaks (cm^{-1})	Vibrational Assignment
620 ~ 776	$\nu(N^+C_3)$, $\nu(CN)$
927	$\nu(N^+C_3)$
965	$\nu(C\text{-}C)$, $\nu(C\text{-}O)$
1077 ~ 1114	$\rho(CH_3)$
1150	$\rho(CH_3)$, $\omega(CH_2)$
1331	$\tau(CH_2)$
1402	$\varsigma(CH_3)$
1508	$\varsigma(CH_3)$, $\varsigma(CH_2)$

Supplementary Figure 9. The measured SERS spectrum and vibrational assignments of acetylcholine in the ss-SERS, compared with the SERS. The ss-SERS peak intensity of acetylcholine at 1150 cm^{-1} corresponding to CH_3 rocking and CH_2 wagging is measured to be maximized more than one thousand times than the SERS at 1 mM in saline solution. (The output power of the laser: 25 mW, the power at the sample: 1 mW, accumulation time: 10 sec)

δ, bending; τ, twisting; ω, wagging; ν, stretching; ρ, rocking.

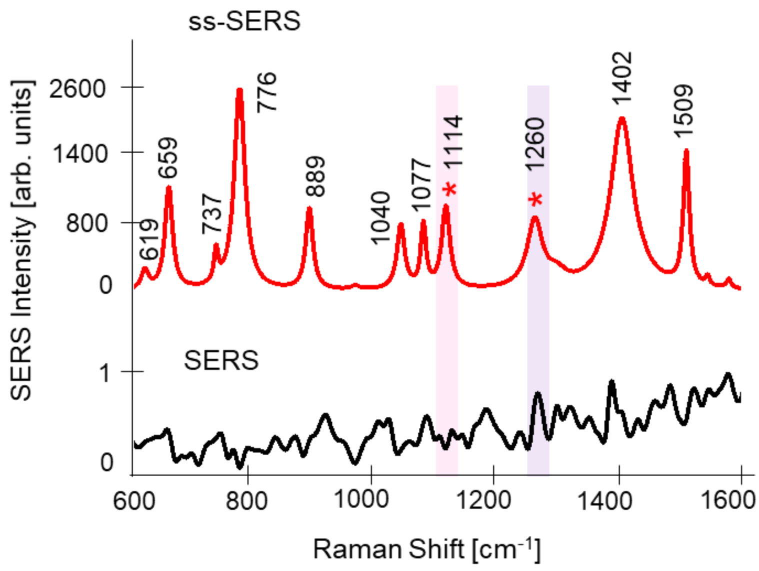

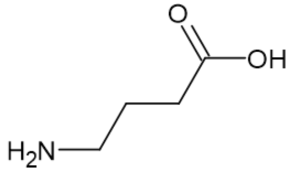

Peaks (cm^{-1})	Vibrational Assignment
619 ~ 776	ς(COO^-)
889 ~ 1040	$\rho(CH_2)$
1077	$\rho(NH_3^+)$, ν(C-N)
1114	$\rho(CH_2)$
1260 ~ 1402	$\omega(CH_2)$
1509	ς(COO^-)

Supplementary Figure 10. The measured SERS spectrum and vibrational assignments of GABA in the ss-SERS, compared with the SERS. Intense bands are seen in the 700 to 1400 cm^{-1} range in the ss-SERS that are recorded at 776, 889, 1040, 1077, 1114, 1260, 1402 cm^{-1}, respectively. The strongest band, which is recorded at 779 cm^{-1} is assigned to the COO^- deformation. Intensity at 779 cm^{-1} is measured to be maximized more than three thousand times than the SERS at 1 mM in saline solution. (The output power of the laser: 25 mW, the power at the sample: 1 mW, accumulation time: 10 sec)

δ, bending; τ, twisting; ω, wagging; ν, stretching; ρ, rocking.

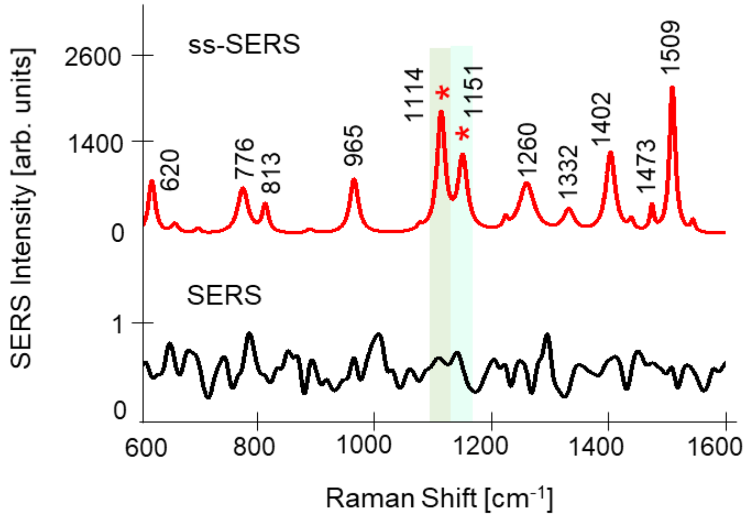

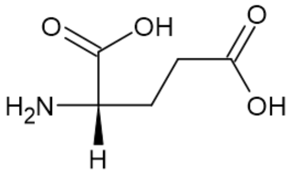

Peaks (cm^{-1})	Vibrational Assignment
620 ~ 776	$\delta(COO^-)$
813	$\rho(CH_2)$
965	$\delta(COO^-)$
1114	$\rho(CH_2)$
1151	$\tau(CH_2)$
1260	$\omega(CH_2)$
1332	$\delta(CH_2)$
1402	$v(COO^-)$
1509	$\delta(NH_3^+)$

Supplementary Figure 11. The measured SERS spectrum and vibrational assignments of glutamate in the ss-SERS, compared with the SERS. The strongest band, which is recorded at 1114 cm^{-1} is assigned to the CH_2 rocking. Intensity at 1114 cm^{-1} is measured to be maximized more than two thousand times than the SERS at 1 mM in saline solution. (The output power of the laser: 25 mW, the power at the sample: 1 mW, accumulation time: 10 sec)

δ, bending; τ, twisting; ω, wagging; v, stretching; ρ, rocking.

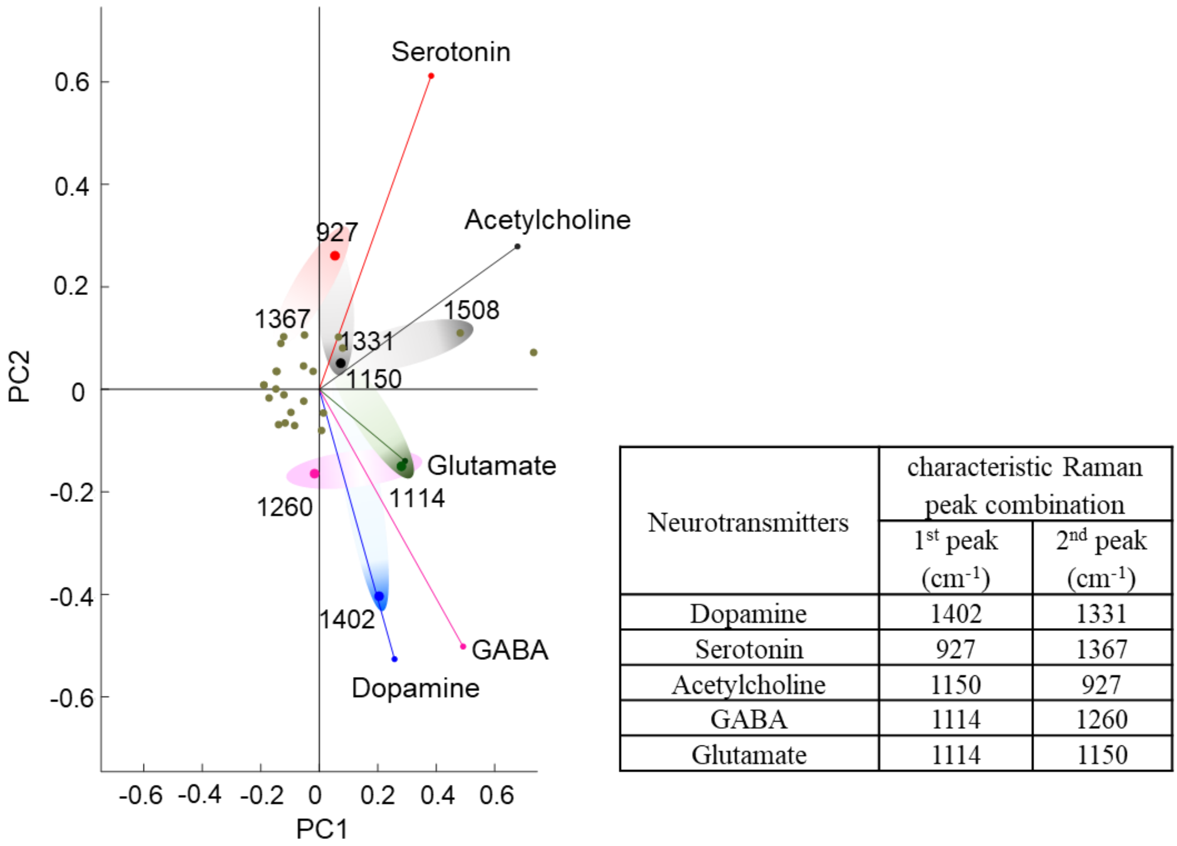

Neurotransmitters	characteristic Raman peak combination	
	1st peak (cm^{-1})	2nd peak (cm^{-1})
Dopamine	1402	1331
Serotonin	927	1367
Acetylcholine	1150	927
GABA	1114	1260
Glutamate	1114	1150

Supplementary Figure 12. Extraction of characteristic Raman peak combination for neurotransmitters of dopamine, serotonin, acetylcholine, GABA, and glutamate. The characteristic Raman peak combination consists of two peaks that have the largest correlation with a specific neurotransmitter in the PCA biplot.

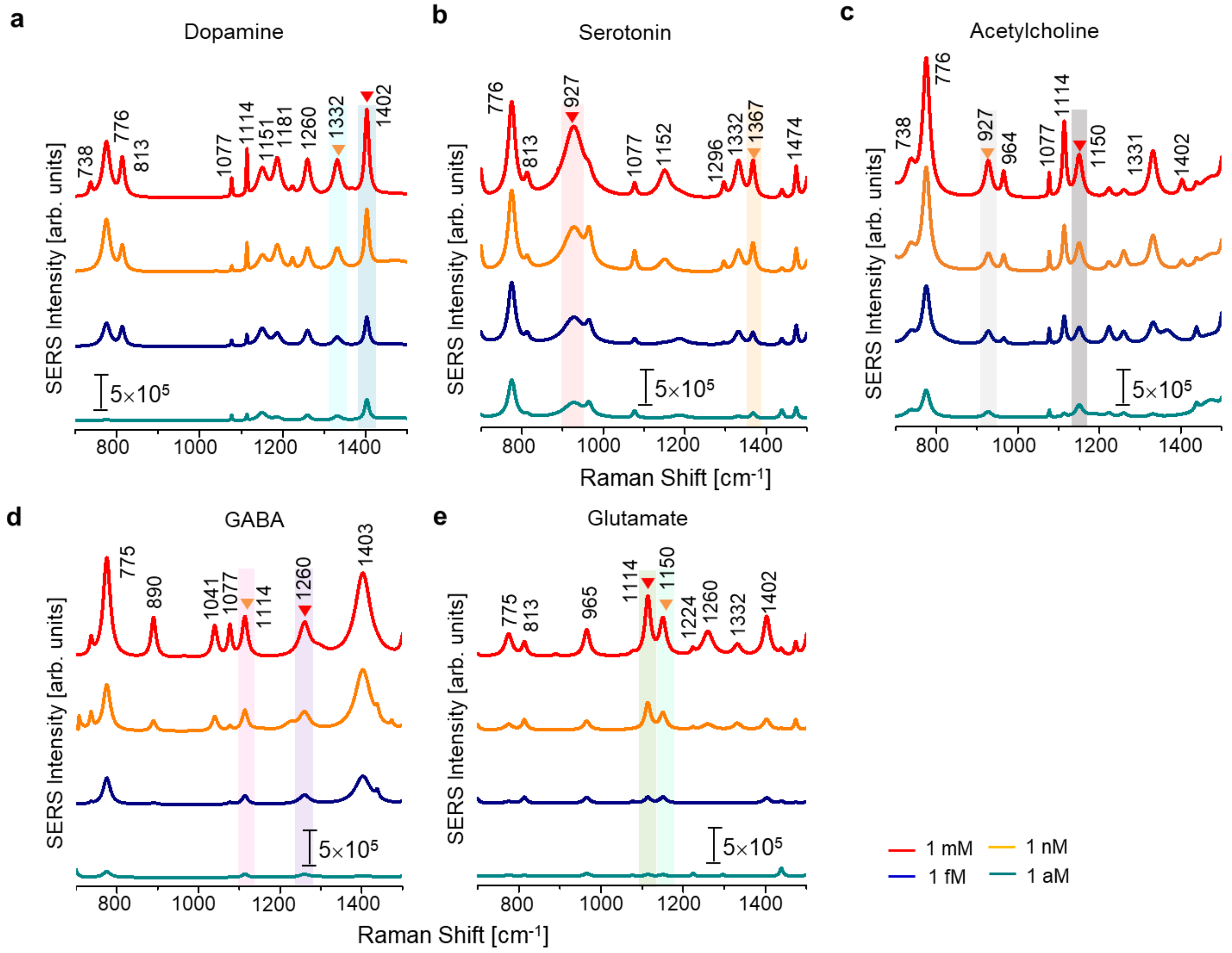

Supplementary Figure 13. ss-SERS spectra of primary neurotransmitters for different concentrations ranging from 1 mM (10^{-3} M) to 1 aM (10^{-18} M). The major SERS peak (1402 cm^{-1}, CH wagging) of dopamine and the major SERS peak (927 cm^{-1}, out of phase breathing) of serotonin decrease as the concentration decreases from 1 mM to 1 aM. In addition, the major SERS peak (1260 cm^{-1}, CH_2 wagging) of GABA and the major SERS peak (1114 cm^{-1}, CH_2 rocking) of glutamate decrease as the concentration decreases from 1 mM to 1 aM. (The output power of the laser: 25 mW, the power at the sample: 1 mW, accumulation time: 10 sec)

δ, bending; τ, twisting; ω, wagging; v, stretching; ρ, rocking.

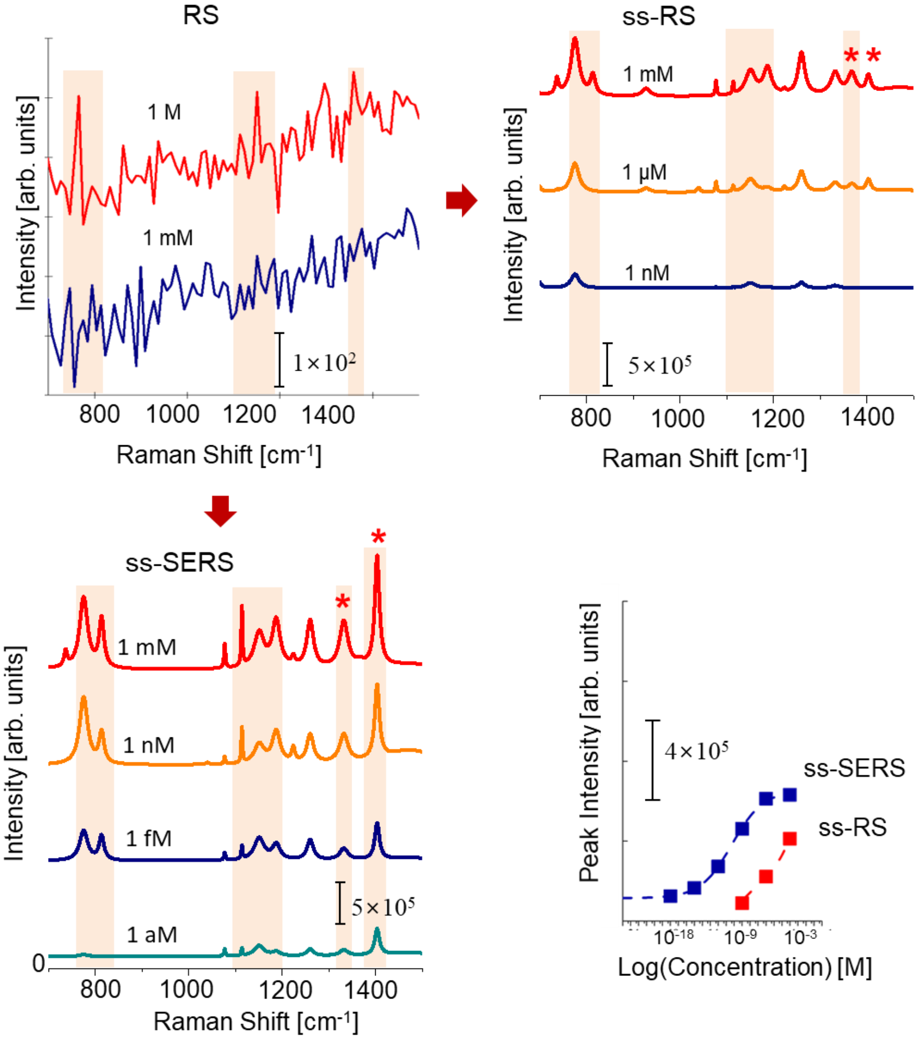

Supplementary Figure 14. SNR enhancement for ss-RS of dopamine at different concentration from 1 mM to 1 nM, compared with results of ss-SERS. The experimental results demonstrate label-free Raman detection of dopamine at nanomolar level as well as 10^3 enhancement in SNR. (The output power of the laser: 25 mW, the power at the sample: 1 mW, accumulation time: 10 sec)

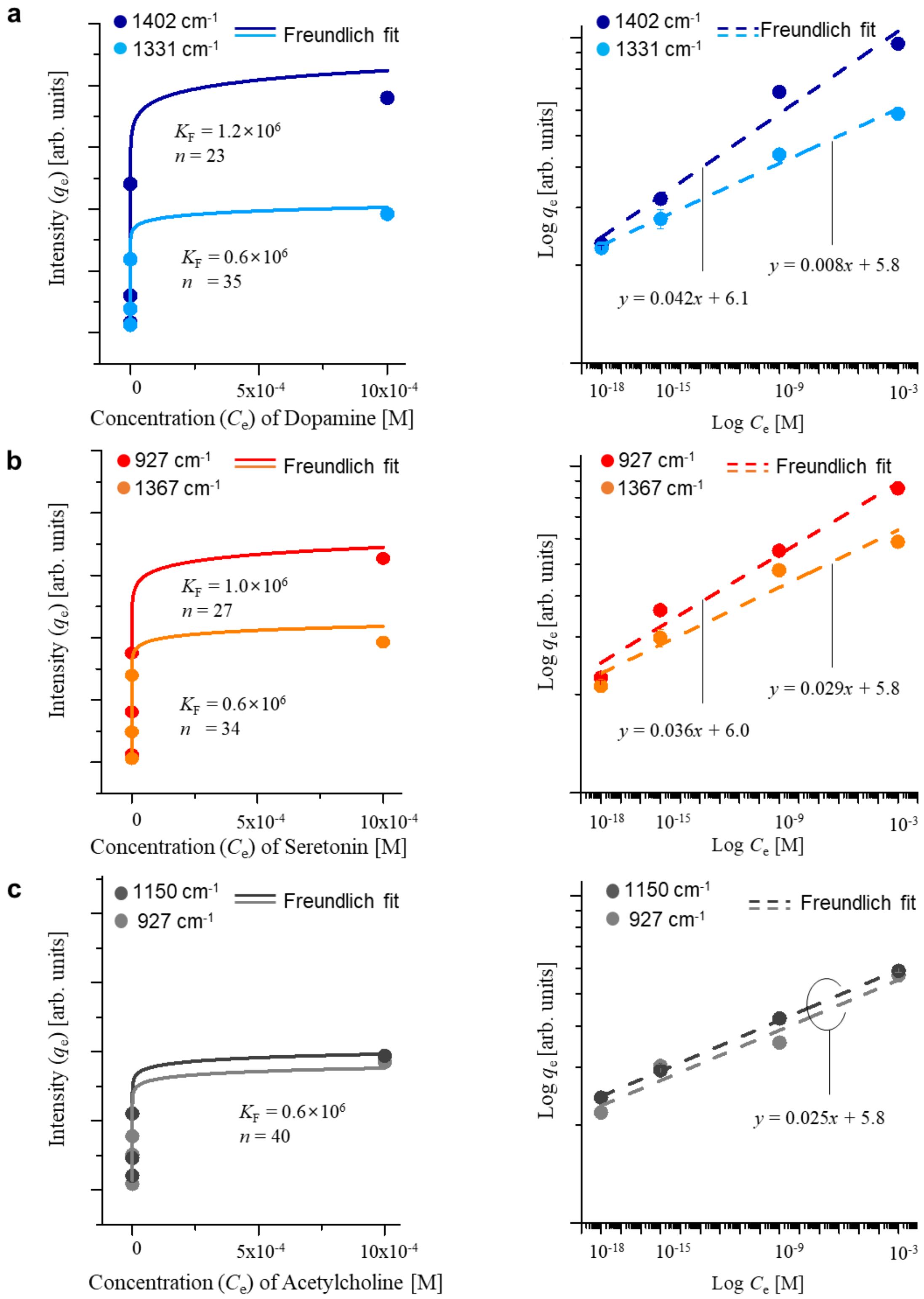

a
1402 cm-1
1331 cm-1
Freundlich fit
$K_F = 1.2\times10^6$
$n = 23$
$K_F = 0.6\times10^6$
$n = 35$
Intensity (q_e) [arb. units]
0
5x10-4
10x10-4
Concentration (C_e) of Dopamine [M]
1402 cm-1
1331 cm-1
Freundlich fit
Log q_e [arb. units]
$y = 0.042x + 6.1$
$y = 0.008x + 5.8$
10-18
10-15
10-9
10-3
Log C_e [M]
b
927 cm-1
1367 cm-1
Freundlich fit
$K_F = 1.0\times10^6$
$n = 27$
$K_F = 0.6\times10^6$
$n = 34$
Intensity (q_e) [arb. units]
0
5x10-4
10x10-4
Concentration (C_e) of Seretonin [M]
927 cm-1
1367 cm-1
Freundlich fit
Log q_e [arb. units]
$y = 0.036x + 6.0$
$y = 0.029x + 5.8$
10-18
10-15
10-9
10-3
Log C_e [M]
c
1150 cm-1
927 cm-1
Freundlich fit
$K_F = 0.6\times10^6$
$n = 40$
Intensity (q_e) [arb. units]
0
5x10-4
10x10-4
Concentration (C_e) of Acetylcholine [M]
1150 cm-1
927 cm-1
Freundlich fit
Log q_e [arb. units]
$y = 0.025x + 5.8$
10-18
10-15
10-9
10-3
Log C_e [M]

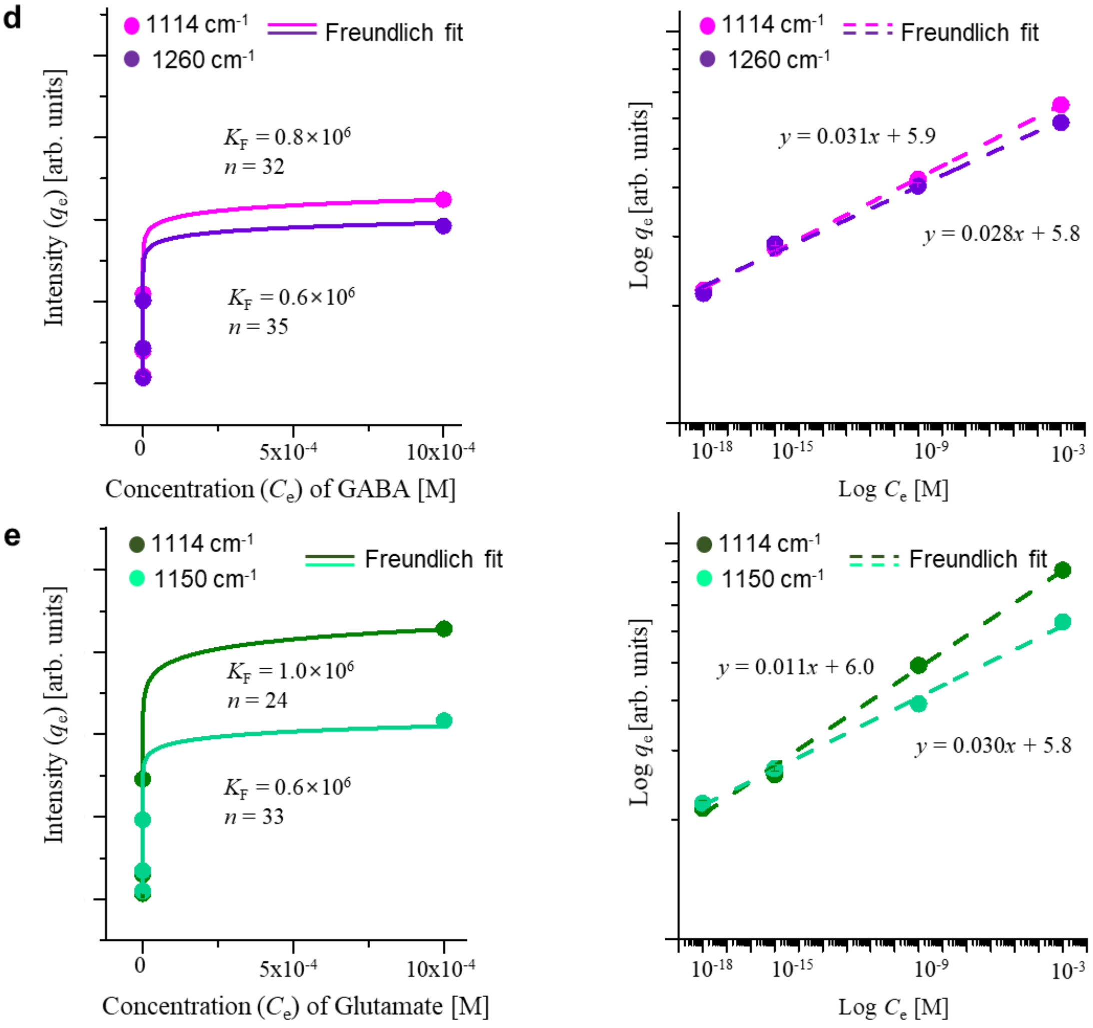

Supplementary Figure 15. LOD curve fitting using Freundlich Isotherm for (a) dopamine, (b) serotonin, (c) acetylcholine, (d) GABA, and (e) glutamate. The concentration range (x-axis) and intensity range (y-axis) are plotted in logarithmic scale to display in detail data at the very low concentration. The nonlinear fit appears as a dotted curve in the plot and are fitted to the Freundlich isotherm model. The isotherms present a linear relationship between ss-SERS intensity and concentrations of the primary neurotransmitters. Freundlich isotherm constants (K_F) of dopamine, serotonin, acetylcholine, GABA, and glutamate show 1.2x10^6, 1.0x10^6, 0.6x10^6, 0.8x10^6, 1.0x10^6, respectively. K_F is an indicator of adsorption capacity, so higher the maximum capacity, higher the K_F.

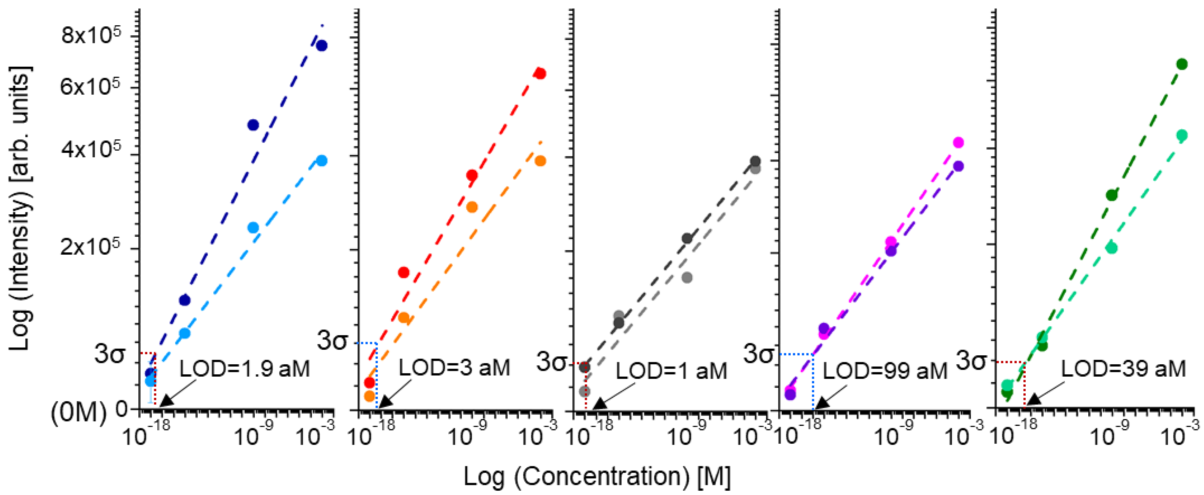

Supplementary Figure 16. LOD calculation for the primary neurotransmitters at the characteristic Raman peaks. In the calibrated ss-SERS spectra where the value of the blank sample (0 M) is removed, the values of a blank sample (0 M) at the characteristic Raman peaks are zero and standard deviation noises (σ) of the peaks at 1402 cm^{-1}, 927 cm^{-1}, 1150 cm^{-1}, 1260 cm^{-1}, and 1114 cm^{-1} are 16816, 20031, 14646, 18268, and 13258, respectively.

Supplementary Table 1. Vibrational assignments for all measured ss-SERS and ss-RS peaks of R6G.

Measured SERS Peaks (cm^{-1})	Measured RS Peaks (cm^{-1})	Vibrational Assignment
620	620	
	659	
	698	C-C ring in-plane bending in xanthen/phenyl rings
737		
775		
	1002	
1077	1040	
1114	1077	C-H in-plane bending in xanthene/phenyl rings
	1114	
	1296	C-O-C stretching in $COOC_2H_5$ group on phenyl ring
1331	1331	C-C stretching in xanthene ring
1402	1402	
1473		C-N stretching in NHC_2H_5
1508	1508	C-C stretching in xanthene ring

Supplementary Table 2. Comparison between the ss-SERS and modulation-based SERS.

	Methods	Difference	Reference
Modulation-based SERS	Time-resolved gating	Collect scattered photons only within the time gate	E.S. Fotso et.al[1]
	Wavelength modulation	Distinguish the Raman signal from the stationary fluorescence signal	B.B. Praveen et.al[2]
	Lock-in detection	Carry signals into the reference frequency of less noise region	Zurich Instruments[3]
Our work	ss-SERS	Suppress noise by peak autocorrelation and near-zero cross-correlation 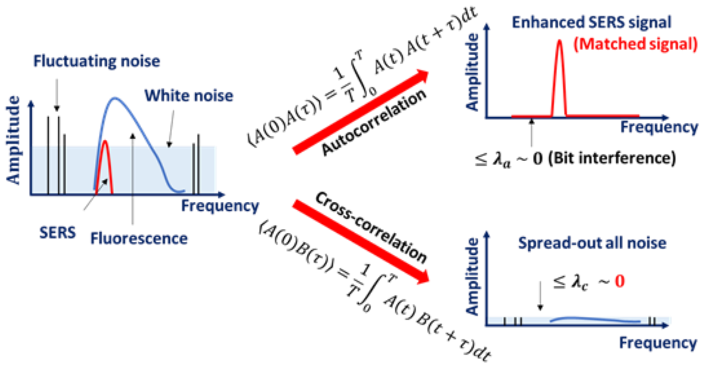	

The ss-SERS is compared with various modulation techniques such as time-resolved gating, wavelength modulation, and lock-in detection that have been developed to remove broad fluorescence noises in Raman spectroscopy. The time-resolved gating is to collect scattered photons only within the time gate corresponding to life time of Raman scattering by using short, intensive laser pulses and time-gated detector and thus to reduce the detection probability of fluorescence photons. The wavelength modulation continuously modulates the laser excitation wavelength to a very short period corresponding to the lifetime of the Raman signal and then distinguish the Raman signal from the stationary fluorescence signal using multianalyte techniques such as PCA. The lock-in detection carries signals into the reference frequency of less noise region by multiplying the signals with a reference signal of a sine wave, and then to isolate the signals from all other frequency components by applying an adjustable low-pass filter. The spread spectrum SERS (ss-SERS) overcomes the limitations of the modulation techniques by using spread out all background noise close to zero by encoding and decoding based on the near-zero cross-correlation of orthogonal codes.

Supplementary Table 3. Empirical formula and simple equation for the Freundlich isotherm and Langmuir isotherm.

Model Isotherm	Empirical Formula	Simple Equation
Freundlich isotherm	$q_\mathrm{e} = K_\mathrm{F} C_\mathrm{e}^{1/n}$	$\log q_\mathrm{e} = \log K_\mathrm{F} + \frac{1}{n}\log C_\mathrm{e}$
Langmuir isotherm	$q_\mathrm{e} = \frac{q_\mathrm{m} \cdot K_\mathrm{L} \cdot C_e}{1 + K_\mathrm{L} \cdot C_e}$	$\frac{1}{q_\mathrm{e}} = \frac{1}{K_\mathrm{L} q_\mathrm{m}}\left(\frac{1}{C_\mathrm{e}}\right) + \frac{1}{q_\mathrm{m}}$

K_F: Freundlich isotherm constant (dimensionless)
n: Freundlich isotherm exponent (dimensionless)
K_L: Langmuir constant (L mg^{-1})
q_m: Maximum adsorption capacity (mg g^{-1})

1 E.S. Fotso Gueutue, A. C., P. Simon, N. Raimboux, L. Hennet, M. R. Ammar. Nanosecond time resolved Raman spectroscopy for solving some Raman problems such as luminescence or thermal emission. *Journal of Raman Spectroscopy*, 1-8 (2018).
2 Bavishna B. Praveen, C. S., Michael Mazilu, Kishan Dholakia, Sumeet Mahajan. Wavelength modulated surface enhanced (resonance) Raman scattering for background-free detection. *Analyst* **138**, 2816-2820 (2013).
3 Principles of lock-in detection and the state of the art. (Zurich Instruments, 2016).
